# Cucurbitacin I Inhibits Cell Motility by Indirectly Interfering with Actin Dynamics

**DOI:** 10.1371/journal.pone.0014039

**Published:** 2010-11-24

**Authors:** David A. Knecht, Rebecca A. LaFleur, Alem W. Kahsai, Christian E. Argueta, Anwar B. Beshir, Gabriel Fenteany

**Affiliations:** 1 Department of Molecular and Cell Biology, University of Connecticut, Storrs, Connecticut, United States of America; 2 Department of Chemistry, University of Connecticut, Storrs, Connecticut, United States of America; University of Birmingham, United Kingdom

## Abstract

**Background:**

Cucurbitacins are plant natural products that inhibit activation of the Janus kinase 2 (JAK2)/signal transducer and activator of transcription 3 (STAT3) pathway by an unknown mechanism. They are also known to cause changes in the organization of the actin cytoskeleton.

**Methodology/Principal Findings:**

We show that cucurbitacin I potently inhibits the migration of Madin-Darby canine kidney (MDCK) cell sheets during wound closure, as well as the random motility of B16-F1 mouse melanoma cells, but has no effect on movement of *Dictyostelium discoideum* amoebae. Upon treatment of MDCK or B16-F1 cells with cucurbitacin I, there is a very rapid cessation of motility and gradual accumulation of filamentous actin aggregates. The cellular effect of the compound is similar to that observed when cells are treated with the actin filament-stabilizing agent jasplakinolide. However, we found that, unlike jasplakinolide or phallacidin, cucurbitacin I does not directly stabilize actin filaments. In *in vitro* actin depolymerization experiments, cucurbitacin I had no effect on the rate of actin filament disassembly at the nanomolar concentrations that inhibit cell migration. At elevated concentrations, the depolymerization rate was also unaffected, although there was a delay in the initiation of depolymerization. Therefore, cucurbitacin I targets some factor involved in cellular actin dynamics other than actin itself. Two candidate proteins that play roles in actin depolymerization are the actin-severing proteins cofilin and gelsolin. Cucurbitacin I possesses electrophilic reactivity that may lead to chemical modification of its target protein, as suggested by structure-activity relationship data. However, mass spectrometry revealed no evidence for modification of purified cofilin or gelsolin by cucurbitacin I.

**Conclusions/Significance:**

Cucurbitacin I results in accumulation of actin filaments in cells by a unique indirect mechanism. Furthermore, the proximal target of cucurbitacin I relevant to cell migration is unlikely to be the same one involved in activation of the JAK2/STAT3 pathway.

## Introduction

Cucurbitacins are plant triterpenoids with a number of interesting biological properties. They possess antiproliferative and/or cytotoxic activity against various cancer cells (for a review, see [1]) and anti-inflammatory activity [2], [3], [4], [5], [6]. Cucurbitacins selectively inhibit the activation of the Janus kinase 2 (JAK2)/signal transducer and activator of transcription 3 (STAT3) signal transduction pathway but does not directly bind and inhibit these proteins [7], [8], [9], [10], [11], [12], [13]. Cucurbitacins have been shown to cause considerable changes in the organization of the actin cytoskeleton in cells, including disruption or rearrangement of normal actin networks and formation of abnormal actin aggregates [10], [14], [15], [16], [17], [18], [19], [20], [21], [22]. In addition, cucurbitacins can affect cell adhesion [23].

While screening the National Cancer Institute (NCI) Diversity Set, we identified cucurbitacin I (NSC 521777; JSI-124; elataricin B) as a potent inhibitor of cell migration in a Madin-Darby canine kidney (MDCK) cell wound closure assay. Cucurbitacin I has also been shown to inhibit the migration of keloid fibroblasts [24], [25]. Assembly and disassembly of actin filaments is required for cell migration, and compounds that affect polymerization of actin or the stability of actin filaments inhibit the motility of cells (for reviews, see [26], [27]). This may be the mechanism by which cucurbitacin I inhibits the movement of cells, but it is as yet unknown how cucurbitacin I mediates its effects on the cytoskeleton. While one report provides evidence that cucurbitacin E may directly stabilize existing actin filaments *in vitro* [20], it is far from clear if this alone accounts for the full extent of dramatic actions of cucurbitacins on the actin cytoskeleton in the cell.

We show that cucurbitacin I inhibits the migration of MDCK cells and B16-F1 mouse melanoma cells, although it has no effect on *Dictyostelium discoideum* cells. Cucurbitacin I caused actin aggregation in MDCK and B16-F1 cells, yet had no direct effect on purified actin polymerization or depolymerization *in vitro*. Cucurbitacins A, B, and C lacked anti-migratory activity against MDCK cells, although they still possessed cytotoxic activity. These results indicate that in cells, cucurbitacin I has an indirect effect on actin dynamics that leads to an over-accumulation of actin, most likely by interfering with actin depolymerization.

## Results

**Figure 1:**
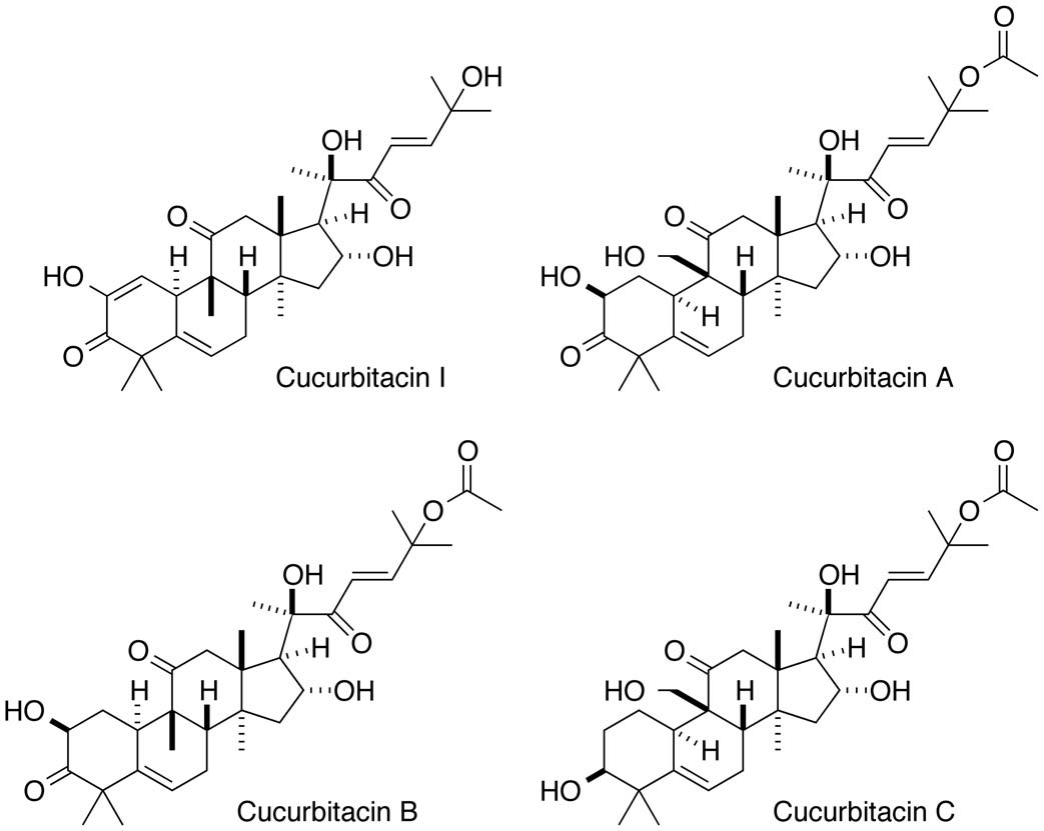
Structures of cucurbitacins examined in this study.

**Figure 2:**
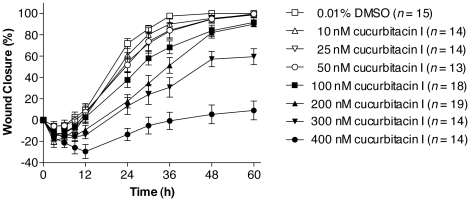
Cucurbitacin I inhibits cell sheet migration during wound closure of MDCK epithelial cell monolayers in a dose-dependent manner. The progress of wound closure in MDCK cell monolayers was followed in the presence of different concentrations of cucurbitacin I. Values represent the mean with standard error of the mean for the indicated number of wounds.

A high throughput wound closure assay with MDCK cells was used to screen the NCI Diversity Set for inhibitors that slowed the rate of cell migration [28]. The most potent hits were evaluated for cytotoxicity, and those with clear subtoxic activity were investigated further. Cucurbitacin I (NSC 521777; structure in Figure 1) was among these compounds (Figure 2 and Table 1). In contrast, cucurbitacins A, B, and C displayed no clear subtoxic antimigratory activity, although they were cytotoxic at higher concentrations (Table 1).

**Table 1:** Activity of cucurbitacins in MDCK cells.

Compound	IC_50_ a	95% CI^b^	MIC^c^	MLC^d^
Cucurbitacin I(NSC 521777)	151 nM	97 nM–234 nM	50 nM	500 nM
Cucurbitacin A(NSC 94743)	NA	NA	500 nM	2,000 nM
Cucurbitacin B(NSC 49451)	NA	NA	500 nM	1,000 nM
Cucurbitacin C(NSC 94744)	NA	NA	500 nM	1,000 nM

a Half-maximal inhibitory concentration (IC_50_) values relative to the maximal response were calculated for inhibition of wound closure at 24 h post-wounding from data for the range of subtoxic concentrations.

b 95% confidence interval (CI).

c Minimum inhibitory concentration (MIC).

d Minimal lethal concentration (MLC).

**Figure 3:**
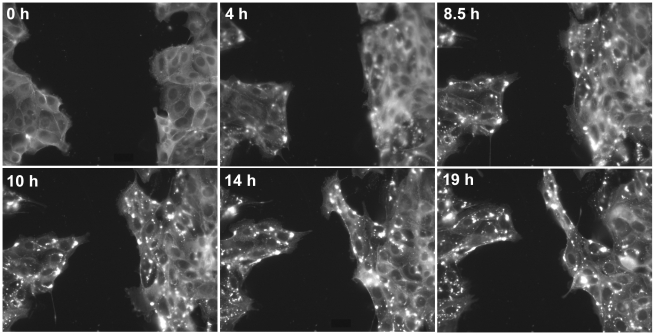
Cucurbitacin I affects the morphology of the wound edge and actin cytoskeletal organization in MDCK cell monolayers. mCherry-actin-expressing MDCK cells were grown to confluence and the monolayer was then wounded. Images of the wound edge every 5 min for 5 h without compound and then for 19 h in the presence of 200 nM cucurbitacin I. Wound closure ceased following addition of the compound and the cells began to accumulate actin aggregates. Time “0 h” in this figure corresponds to the time of compound addition, which is 5 h in Movie S1.

We imaged the wound closure process in MDCK cells expressing mCherry-actin by time-lapse fluorescence microscopy (Figure 3 and Movie S1). The wound closed steadily at about 0.45 µm/min for 2 h in the absence of compound. Upon addition of the compound, wound edge translocation was greatly inhibited, and actin aggregates began to accumulate in the cells. By 2 h after addition of compound, the rate of closure decreased to 0.1 µm/min. After several more hours, the wound edge again began to slowly move into the open area; however, instead of maintaining a consistent front, some areas advanced while others retracted as if the sheet had lost adhesive continuity.

**Figure 4:**
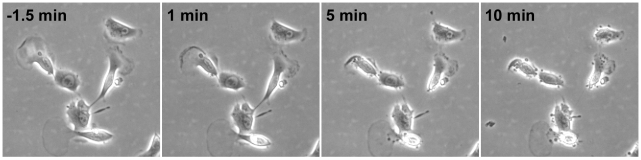
Cucurbitacin I inhibits motility of B16-F1 melanoma cells. Phase-contrast images of migrating B16-F1 cells were captured every 30 s on a laminin-coated dish. 200 nM cucurbitacin I was then added to the chamber. The panels show representative time points before and after compound addition with “0 h” being when compound was added.

**Figure 5:**
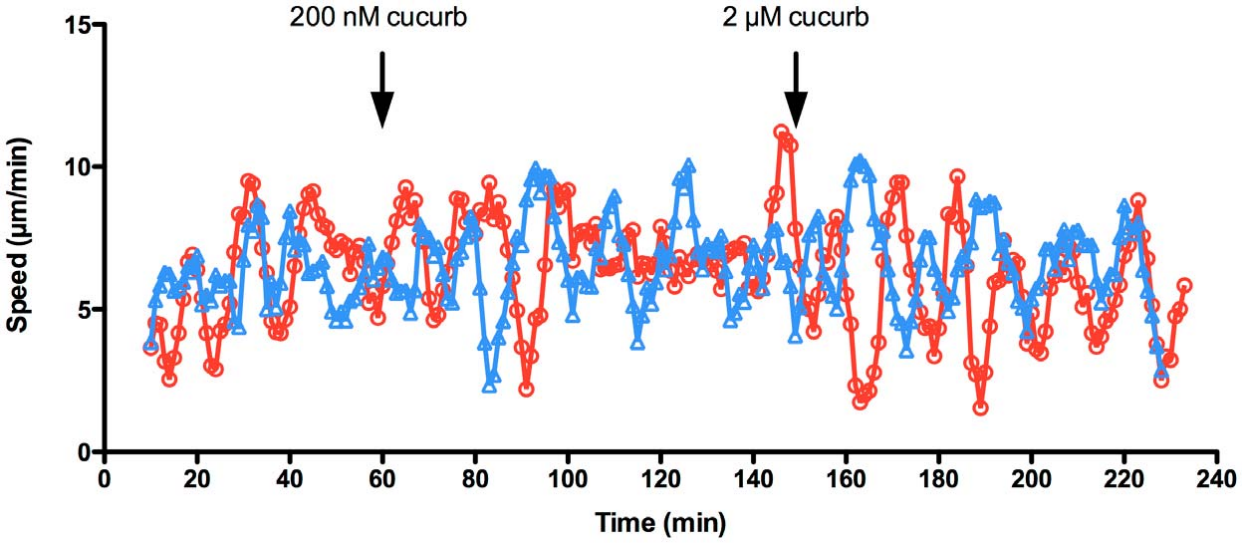
Cucurbitacin I does not affect the motility of *Dictyostelium* amoebae. *Dictyostelium* cells were plated in growth medium and allowed to attach for 1 h. Images were captured every 15 s for 1 h, and then cucurbitacin I was added to 200 nM. Imaging was continued for 1.5 h and then the concentration of cucurbitacin I was raised to 2 µM and imaging continued for another 1.5 h. The graph shows the smoothed speeds of two representative cells from Movie S4). There was no significant difference between the mean speed of the population of cells in the presence or absence of the compound (control, 6.2±3.1 µm/min; 200 nM cucurbitacin I, 6.3±3.5 µm/min; 2 µM cucurbitacin I, 6.0±3.2 µm/min; *n* = 6 cells for all treatments).

We next looked at the effect of cucurbitacin I on motile B16-F1 melanoma cells and *Dictyostelium discoideum* amoebae. When 200 nM cucurbitacin I was applied to migrating B16-F1 cells, translocation rapidly ceased (Movies S2 and S3). After 45 min, the compound was washed out, but it took about an hour before the first cells began to regain movement. Full recovery of the population occurred gradually over the next 5 h. When imaged at higher temporal and spatial resolution, lamellipodial ruffling and protrusion stopped within 30 s of treatment with cucurbitacin I, and within 1 min the lamellipodia began to retract (Figure 4 and Movie S3). Within 10 min, the cells had become rounded and small blebs appeared around the periphery. We then tested the effect of cucurbitacin I on the motility of *Dictyostelium* amoebae. No significant change in average speed or cell morphology were seen at concentrations as high as 2 µM cucurbitacin I (Figure 5 and Movie S4).

**Figure 6:**
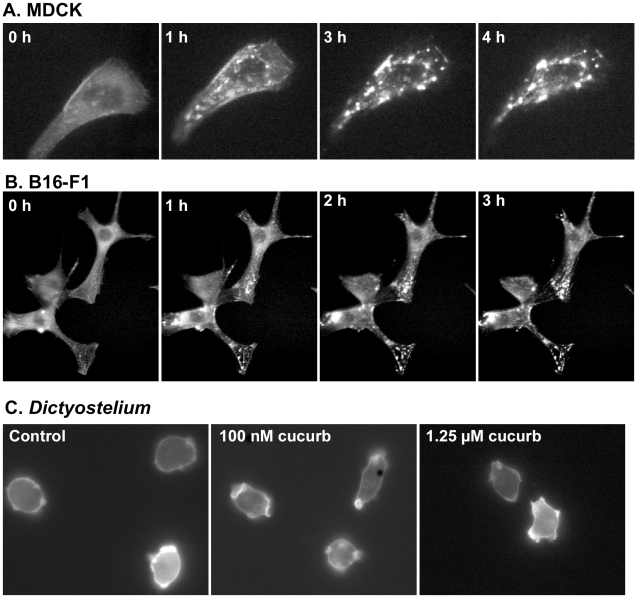
Cucurbitacin I causes actin aggregation in MDCK and B16-F1 cells. (A) mCherry-actin-expressing MDCK cells were plated at low density and then imaged before (0 h) and after addition of 200 nM cucurbitacin I. Images were collected every 5 min for 4.5 h. Cells cease to move and then begin to accumulate actin aggregates within 1 h of compound addition. (B) mCherry-actin-expressing B16-F1 cells were treated with 25 nM cucurbitacin I. The cells ceased to move and actin aggregates began to accumulate within 1 h of compound addition. (C) *Dictyostelium* cells expressing the F-actin probe dRFP-FilABD were plated in Petri dishes at low density in HL5 growth media and allowed to settle for several hours. Cells were then placed in 1 mL of medium containing: (A) 0.25% DMSO; (B) 100 nM cucurbitacin I; (C) 1.25 µM cucurbitacin I. Cells were incubated overnight at 21°C and then imaged. No aggregate formation or other alterations of the actin cytoskeleton were observed.

**Figure 7:**
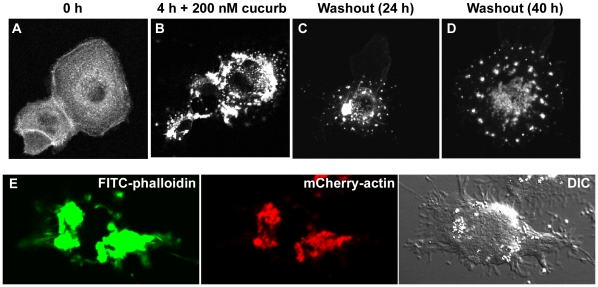
Actin aggregates persist after removal of cucurbitacin I. B16-F1 cells expressing mCherry-actin were imaged before (A) and after 4 h in the presence of 200 nM cucurbitacin I (B). The compound was then washed out and imaging continued. Images are shown at (C) 24 h and (D) 40 h after washout. Each panel is a maximum-intensity projection of 7 confocal *Z* sections through a representative cell. (E) After 4 h in cucurbitacin I, one sample of B16-F1 cells was fixed and stained with fluorescein-conjugated phalloidin to visualize F-actin. A single confocal *Z* slice showing the aggregates stained with fluorescein-phalloidin (left panel), mCherry-actin (center panel) is shown along with a differential interference contrast (DIC) image (right panel).

Having shown that cucurbitacin I has a rapid and reversible effect on cellular motility in MDCK and B16-F1 cells, changes in the localization of actin-containing structures were examined in compound-treated cells expressing mCherry-actin. During wound closure in MDCK cell monolayers, punctate fluorescent structures appeared in the cytoplasm after addition of cucurbitacin I (Figure 3). In low-density MDCK cell cultures, mCherry-actin produced a diffuse signal throughout the cytoplasm, with more intense localization in areas where rapid actin polymerization was occurring, such as in new protrusions (Figure 6A and Movie S5). Upon addition of 200 nM cucurbitacin I, cells ceased lamellar extension, and small punctate aggregates began to form throughout the cytoplasm (Figure 6A and Movie S5). The aggregates became larger over time and the diffuse cytoplasmic signal decreased, suggesting a shift from actin monomer (globular actin; G-actin) to actin polymer (filamentous actin; F-actin). Similar results were obtained when B16-F1 cells expressing mCherry-actin were treated with the compound (Figure 6B). In cells treated with 200 nM cucurbitacin I for 4 h, aggregates persisted for days after the compound was washed out (Figure 7). To demonstrate that the aggregates are formed from F-actin rather than G-actin, cells treated with cucurbitacin I for 4 h were fixed and stained with FITC-phalloidin, which only binds F-actin; the aggregates that were labeled with mCherry-actin were also stained by FITC-phalloidin (Figure 7E). Cucurbitacin I did not cause actin aggregates to form in *Dictyostelium* amoebae (Figure 6C).

**Figure 8:**
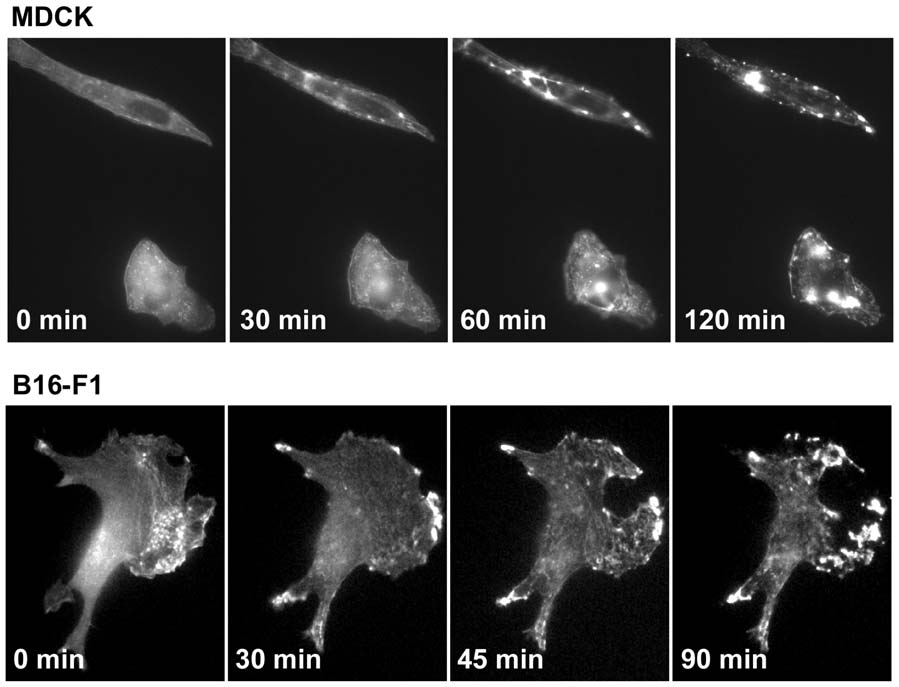
Jasplakinolide inhibits cell motility and causes actin aggregates to form. (A) MDCK cells were plated at low density and imaged as in Figure 5A but in the presence of 200 nM jasplakinolide. The cells cease to move and actin aggregates begin forming within 1 h of compound addition. (B) B16-F1 cells were imaged as in Figure 5B but in the presence of 200 nM jasplakinolide. Cells were spread and moving before addition of jasplakinolide but ceased to move and accumulated actin aggregates within 1 h of treatment with compound. The cells also showed fragmentation of the lamellipodium, which was not observed in cells treated with cucurbitacin I.

The formation of actin aggregates in cucurbitacin I-treated cells indicates that the compound is having an effect on actin depolymerization. In an attempt to learn more about the mechanism by which cucurbitacin I causes actin aggregation, we compared its effects to that of jasplakinolide, a compound that directly binds F-actin and stabilizes the filaments. Treatment of migrating B16-F1 cells with jasplakinolide resulted in cessation of movement within 5 min (Movie S6), as it does in fibroblasts [29]. Within 2 h of compound addition, cells had retracted all processes and became rounded. The cells recovered after compound removal, and after 4 h, they had begun to move again (Movie S6). Jasplakinolide also caused motility to cease and the formation of actin aggregates in MDCK cells (Figure 8A). In B16-F1 cells, jasplakinolide caused the formation of small actin aggregates, but the more dramatic effect was the collapse and fragmentation of the lamellipodium (Figure 8B and Movie S6). This was not observed with treatment with cucurbitacin I, indicating that the effect of the compounds on the actin cytoskeleton is likely to be mechanistically different.

**Figure 9:**
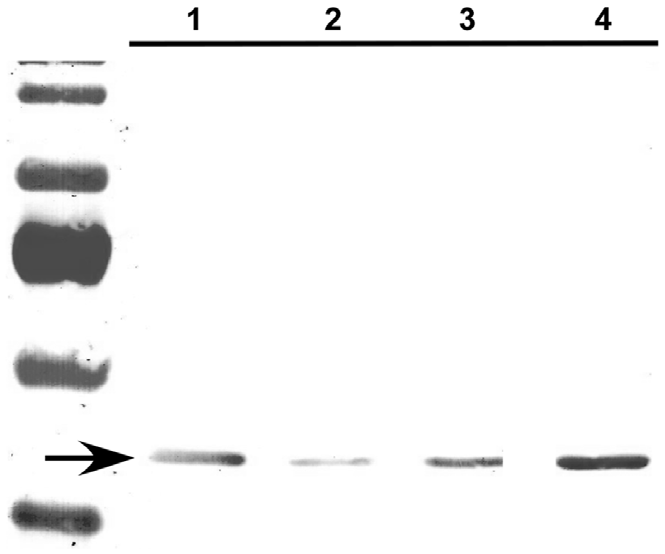
Cucurbitacin I stabilizes actin filaments *in vivo*. Cells were incubated for 2 h with 0.1% DMSO (lanes 1 and 2) or 200 nM cucurbitacin I (lanes 3 and 4), then harvested, lysed and centrifuged to separate G actin (supernatant, lanes 1 and 3) from F-actin (pellet, lanes 2 and 4). Samples were electrophoresed on an SDS-polyacrylamide gel, transferred to a polyvinylidene fluoride membrane and subjected to Western blot analysis with an anti-β-actin antibody. The ratio of F-actin to G-actin increased from 0.19 in the control to 2.5 in the cucurbitacin I-treated cells.

**Figure 10:**
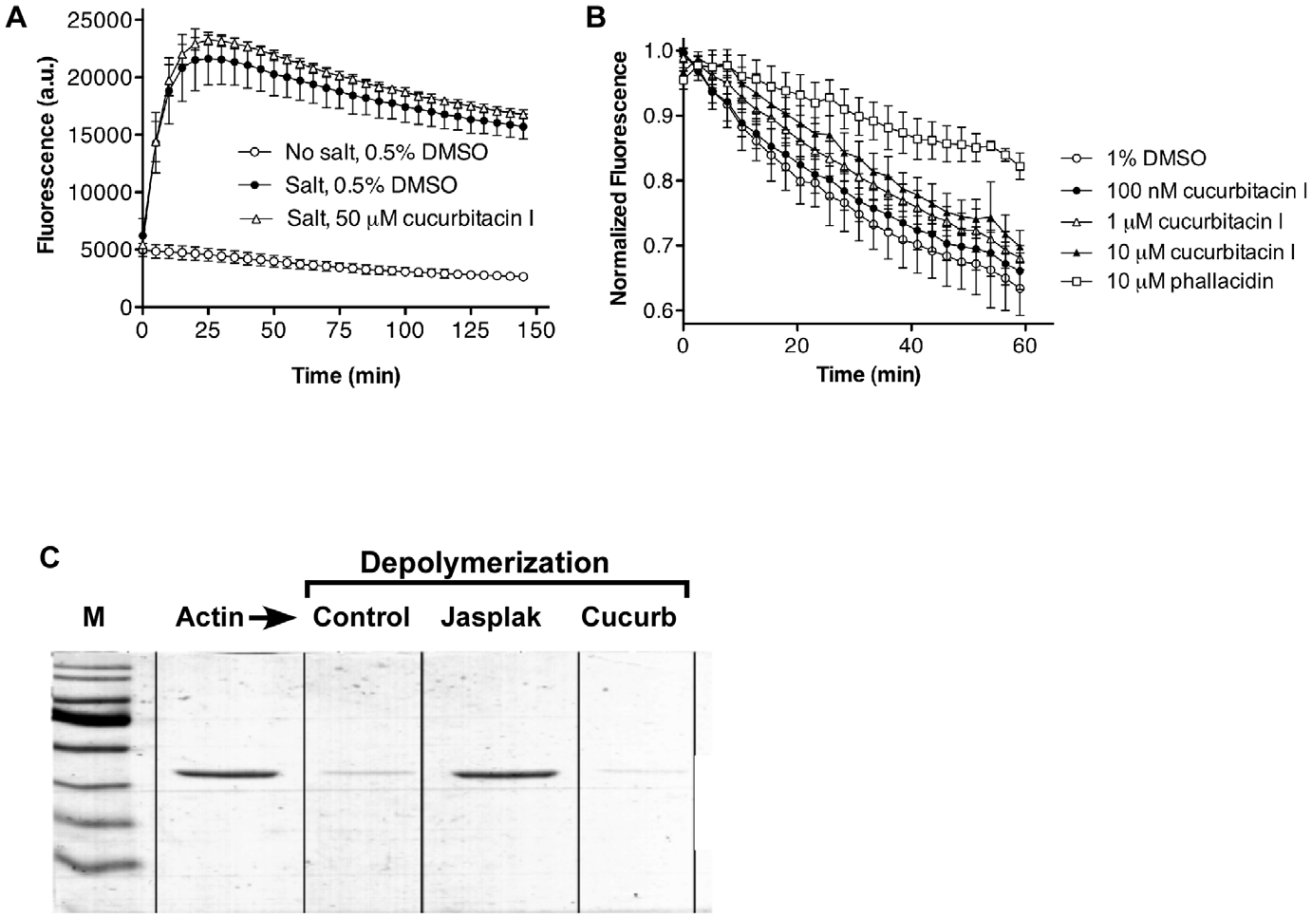
Cucurbitacin I does not directly affect actin polymerization, depolymerization or steady-state levels of F-actin *in vitro*. (A) Pyrene-labeled rabbit muscle G-actin polymerization was measured in the presence or absence of 50 µM cucurbitacin I as described in Materials and Methods. Values represent the mean with standard deviation (SD) for *n* = 4. The compound had no effect on actin polymerization. (B) Pyrene-G-actin was polymerized to pyrene-F-actin and then diluted to induce actin filament disassembly, as described in Materials and Methods, in the presence or absence of cucurbitacin or phallicidin at the indicated concentrations. Phallacidin inhibited actin depolymerization, but cucurbitacin I only weakly delayed the start of depolymerization and only at relatively high concentrations, while not affecting the rate of depolymerization (note similar slopes once depolymerization starts). Values represent the mean with SD for *n* = 9. (C) Cucurbitacin I does not directly stabilize F-actin. Purified human non-muscle G-actin was polymerized to F-actin and then pelleted immediately (lane 1) or after dilution to initiate depolymerization and overnight incubation in the absence (lane 2) or presence of 200 nM jasplakinolide (lane 3) or 200 nM cucurbitacin I (lane 4). Pellets were solubilized in SDS sample buffer, and samples were then electrophoresed on an SDS-polyacrylamide gel and stained with Coomassie blue. Jasplakinolide prevented filament depolymerization, but cucurbitacin I had no effect.

If cucurbitacin I stabilizes F-actin, then this might lead to a change in the ratio of F- to G-actin in cells. In cells treated for 2 h with cucurbitacin I, there was a shift in the F-/G-actin ratio from 0.2 to 2.5 (Figure 9). In order to determine whether cucurbitacin I acts directly on actin filaments or acts indirectly through other actin-binding proteins, the effect of the compound on purified actin was measured. The rate of actin polymerization from pyrene-G-actin in the presence or absence of cucurbitacin I *in vitro* was indistinguishable (Figure 10A). To test the effect of the compounds on actin filament disassembly, pyrene-G-actin was polymerized to pyrene-F-actin and then diluted to induce depolymerization in the presence or absence of compound. Cucurbitacin I at high concentrations had a weak inhibitory effect on actin depolymerization, but this was much weaker than the known direct actin-stabilizing compound phallacidin (Figure 10B). The effect was also qualitatively different from that of phallacidin. High concentrations of cucurbitacin I seemed to delay the onset of depolymerization, but once the filaments began to disassemble, the rates of depolymerization were similar to the DMSO control (note similar slopes in the presence of cucurbitacin I as in its absence in Figure 10B). Furthermore, in an *in vitro* actin depolymerization assay based instead on pelleting of F-actin, 200 nM cucurbitacin I had no effect on actin depolymerization, whereas 200 nM jasplakinolide prevented depolymerization (Figure 10C).

It is not clear what proteins cucurbitacin I interacts with to prevent actin depolymerization. Two potential candidates are cofilin and gelsolin, both of which have actin-severing activity and appear to play roles in actin depolymerization in cells (for a review, see [30]). The side chains of cucurbitacin I and other cucurbitacins with potent activity in cellular studies contain a potentially reactive α,β-unsaturated ketone (Michael acceptor) that appears important for activity [7], [14], [31] and likely alkylates target proteins. Purified cofilin or gelsolin were treated with cucurbitacin I and subjected to liquid chromatography (LC)-electrospray ionization (ESI)-mass spectrometry (MS), as described in Materials and Methods. Neither protein showed a change in mass after compound treatment (Figure S1) indicating the compound did not bind covalently to either protein.

## Discussion

Previously published data has shown that cucurbitacin I inhibits activation of the JAK2/STAT3 signal transduction pathway but does not appear to directly inhibit JAK2 kinase activity or STAT3's function as a transcription factor [7], [8], [9], [10], [11], [12], [13]. It is likely that cucurbitacin I acts upstream of JAK2 and STAT3 by inhibiting some as-yet undefined component of this pathway or that the compound indirectly affects the pathway by inhibiting some factor that may feed into or modulate the pathway. Cucurbitacins B and D have also been shown to antagonize *Drosophila* steroid hormone binding to the ecdysone receptor [32]. Cucurbitacins have dramatic effects on the organization of the actin cytoskeleton, resulting in alterations in the cell's normal actin networks and formation of actin aggregates [10], [14], [15], [16], [17], [18], [19], [20], [21], [22], although the mechanism by which this occurs is unknown. Cucurbitacins have also been reported to inhibit cell adhesion [23].

We have shown that cucurbitacin I is a potent inhibitor of cell motility that also disrupts normal actin dynamics. It is unknown whether cucurbitacin I's effect on the JAK2 pathway and STAT3-dependent transcription is independent or interdependent of its effects on the actin cytoskeleton. It is well known that altering transcription takes hours to affect the cell. Our data has shown that inhibition of motility and the compound's effects on the cytoskeleton occur within seconds to minutes of compound addition. This suggests that cucurbitacin I's effect on transcription does not cause motility inhibition or cytoskeletal abnormalities. It is possible that cucurbitacin I's disruption of the cytoskeleton may lead to transcriptional effects. It is probable that the two effects are both caused by one upstream target because it is unlikely that cucurbitacin I binds to two different target molecules, one that controls transcription and one that regulates actin.

One of the most striking cellular abnormalities observed following treatment with cucurbitacin I is the formation of large cytoplasmic actin aggregates. After about 1 h of compound incubation, actin structures appear thicker throughout the cytoplasm. Some of these structures look similar to stress fibers. As treatment continues, these fibers seem to condense into aggregates. Once these aggregates have formed, they persist within the cell for several days after compound removal. It is tempting to speculate that these aggregates would cause a defect in cell motility, but that seems not to be the case. Interestingly, the inhibition of motility and formation of aggregates occur at within drastically different time frames over the course of treatment. Inhibition of motility occurs within minutes while aggregate formation does not begin until at least 1 h of treatment. This indicates that the aggregates themselves are not what cause the cells to cease translocation. In fact, our data has shown that cells will recover movement after compound removal while cytoplasmic aggregates are still present. Thus, the inhibition of motility is a short term and reversible effect whereas actin aggregation is a longer-term effect.

Cucurbitacin I does not have any apparent effect on migration of the cellular amoeba *Dictyostelium discoideum*. Despite overnight incubation in 1.2 µM cucurbitacin I, *Dictyostelium* do not show changes in motility or cytoskeletal morphology. In contrast, actin aggregates do form in *Dictyostelium* and other cells following treatment with actin-stabilizing compound jasplakinolide [33], [34]. *Dictyostelium* is often used as model systems for higher organisms because this organism contains many proteins that are orthologous to mammalian proteins. The fact that *Dictyostelium* is not affected by cucurbitacin I suggests that the target of cucurbitacin I does not have a close homolog in *Dictyostelium*. This is consistent with the observation that cucurbitacin I does not directly stabilize actin because actin is highly conserved between *Dictyostelium* and mammalian cells.

In an attempt to further characterize the effects of cucurbitacin I, we compared its action to that of actin-stabilizing compound, jasplakinolide. Aggregates caused by jasplakinolide were visually indistinguishable from those induced by cucurbitacin I. We have found that although both of these compounds have similar downstream effects, they do not act by the same mechanism. While jasplakinolide directly stabilizes F-actin *in vivo* and *in vitro*, cucurbitacin I has only a weak effect *in vitro* and only at high concentrations in the pyrene-actin depolymerization assay, similar to the results reported by Momma *et al.* for cucurbitacin E [20]. Cucurbitacin I's activity in cells is orders of magnitude greater than this weak activity *in vitro.* Moreover, the weak stabilization of actin filaments at high concentrations of cucurbitacin I is qualitatively different from the effects of phallacidin: the initiation of depolymerization is delayed but once depolymerization begins, it appears to do so at the same rate as the control. It is hard to imagine that even with the possibility of sequestration of cucurbitacin I in the cell to high local concentrations, any direct actin-stabilizing activity of cucurbitacin I could explain the highly potent activity of cucurbitacin in cells, nor would it explain cucurbitacin I's ability to selectively inhibit activation of the JAK2/STAT3 pathway. Cucurbitacin I more likely targets other proteins that are involved in signaling and the regulation of actin depolymerization.

Cucurbitacins A, B, and C – all of which have an acetylated side chain in addition to other differences from cucurbitacin I – did not possess subtoxic antimigratory activity against MDCK cells. The potentially reactive Michael acceptor function of the side chain of the most bioactive cucurbitacins in a number of cellular studies appears important for activity [7], [14], [31]. These cucurbitacins may covalently bind target proteins. Two potential candidates whose inhibition would be consistent with the observed activity of cucurbitacin I are the actin-severing and actin-depolymerization-promoting proteins cofilin and gelsolin (for a review, see [30]). We found by LC-MS that cucurbitacin I does not covalently bind cofilin or gelsolin.

Nakashima *et al*. found that the Michael acceptor on the side chain of cucurbitacin E was important for its potent antiproliferative activity, and they pulled down cofilin from cell extracts with a biotinylated derivative of cucurbitacin E, although it is not clear if the interaction is direct or indirect [31]. They showed that cucurbitacin E blocks the inactivating phosphorylation of cofilin, which would be expected to result in increased cellular cofilin activity [31]. The alterations in cellular F-actin that could arise as a result of changing cofilin activity are particularly complex. While increasing cofilin activity would be expected to result in more filament severing, it may or may not result in reduced F-actin content. In fact, severing by cofilin has been proposed to play a major role in the generation of new barbed ends, which would elongate to increase lamellipodial F-actin (for a review, see [35]). Nakashima *et al.* also found decreased F-actin in HT1080 fibrosarcoma cells [31], contrary to our data and those of others [18], [19], [20] demonstrating actin aggregation and increased F-actin in cells treated with cucurbitacins.

Interestingly, Nakashima *et al.* found that cucurbitacin I had much lower potency than cucurbitacin E in inhibition of cofilin phosphorylation and effects on actin [31], which may suggest a different profile of specific cellular targets. Cucurbitacin I and cucurbitacin E differ in that the latter is acetylated (like cucurbitacins A, B, and C) on the terminal alcohol of the side chain that constitutes part of the putative pharmacophore of these molecules. Thus, these two cucurbitacins may have somewhat different target selectivity. While cofilin or an associated protein may be a target of cucurbitacin E, the activities described by Nakashima *et al.* [31] do not explain the F-actin-stabilizing effects of cucurbitacins or their inhibitory effects on the JAK2/STAT3 pathway. Therefore, other direct molecular targets of cucurbitacins remain to be identified. Such a target could be an upstream or feedback component of the JAK2/STAT3 pathway that also regulates the stability of actin filaments, or else the different activities of cucurbitacins may result from the targeting of separate proteins. Given the very rapid kinetics of inhibition of cell motility, the latter possibility appears more likely.

## Materials and Methods

### Compound Preparation and Storage

The NCI Diversity Set and solid samples of cucurbitacins A, B, C, and I were kindly provided by the NCI. Stock solutions of 10 mM cucurbitacins A, B, C, and I in dimethyl sulfoxide (DMSO) were prepared from the desiccated solid samples. Stock solutions were stored in a desiccator at −20°C and used within 2 months of preparation.

### Cell Lines and Vectors

*Dictyostelium discoideum* strain amoebae were grown in HL5 nutrient media [36] at 21°C. To image actin dynamics, cells were transfected with dRFP-FilABD, which behaves similarly to the GFP-FilABD probe previously described [37]. The probe was made by isolating the actin-binding domain of *Dictyostelium* filamin by PCR using forward primer 5′-CAGTAGGATCCATGGCTGCTGCTCCAAGT-3′ and reverse primer 5′-TACGCTCTAGAGGCATCTGAAGTTTC-3′. These primers added BamHI and XbaI sites, allowing the fragment to be ligated into dRFP-pDXA-3H-Hygro [38] into which dimer2 RFP [39] had already been inserted (pDXA-dRFP). MDCK cells, either “normal” or stably transfected cells expressing mCherry [40] fused to human β-actin (mCherry-actin), were grown in Minimum Essential Medium containing 0.1% nonessential amino acids, ampicillin/streptomycin antibiotics, and 10% fetal bovine serum (FBS) or newborn calf serum at 37°C and 5% CO_2_. B16-F1 cells stably expressing mCherry-actin were grown in Dulbecco's Modified Eagle's Medium with D-glucose, L-glutamine, and 10% FBS at 37°C and 5% CO_2_.

### Imaging and Analysis of Mammalian Cell Migration

Wound closure experiments to evaluate migration of MDCK cells in the presence of cucurbitacin I (both initial screening and subsequent quantitative analyses for concentration-response profiling) were carried out as previously described, as were determinations of cell viability at the end of each experiment [28].

For live-cell imaging, MDCK cells (2×10^5^) were plated on Bioptechs chambers (coated with 5 µg/mL laminin) and grown overnight in bicarbonate-free HAMS-F12 medium with 10% FBS at 37°C. The medium was replaced with 500 µL fresh bicarbonate-free HAMS-F12 medium buffered with 10 mM HEPES, and the Bioptechs chambers were placed in a Bioptechs Delta T stage temperature controller set to 37°C. A single scratch wound was created in the monolayer by scraping the surface of the dish with a 200-µL pipet tip. The wound was imaged every 5 min for several hours on an automated Zeiss 200 M inverted microscope with Openlab software (Perkin-Elmer/Improvision), and then cucurbitacin I was added in an equal volume of new medium for a final concentration of 200 µM. Images were taken for several more hours and then analyzed with ImageJ software [41]. The long-term confocal imaging of aggregates in cells treated with cucurbitacin I was performed using a Pathology Devices stage incubator (maintaining 37°C and 5% CO_2_) on a Nikon A1R confocal microscope.

To measure random motility, MDCK or B16-F1 cells were plated on 5 µL/mL laminin-coated Bioptechs chambers at 37°C at low density and allowed to settle overnight. Imaging was done before and after addition of an equal volume of medium containing cucurbitacin I or jasplakinolide to final concentrations indicated in the figure and movie legends. Motility was analyzed with ImageJ software and the MTrackJ plugin.

### Imaging and Analysis of Dictyostelium Cell Motility and Cytoskeleton

For analysis of cell speed, *Dictyostelium* amoebae (strain NC4A2) were plated on plastic Petri dishes in HL5 growth medium and allowed to attach for 1 h. Images were captured every 15 s for 1 h, and then cucurbitacin I was added to 200 nM. Imaging was continued for 1.5 h, and then the concentration of cucurbtacin I was raised to 2 µM and imaging continued for another 1.5 h. The captured images were analyzed with ImageJ software with the mTrackJ plugin to manually track the movements of representative cells. The speeds were analyzed with Graphpad Prism by a Mann-Whitney non-parametric U test.

To determine whether cucurbitacin I causes aggregate formation, cells expressing dRFP-FilABD were plated in glass-bottom Petri dishes (Willco Wells) and treated with compound overnight in HL medium. dRFP-FilABD like GFP-FilABD [37] associates with F-actin-containing structures in cells, including actin aggregates induced by jasplakinolide (data not shown). The cells were imaged with a 100× oil immersion objective.

### Determination of G- and F-Actin Levels in Control and Compound-Treated Cells

Subconfluent MDCK cells were treated overnight with 0.1% DMSO or 200 nM cucurbitacin I. Cells were harvested into 1.5 mL tubes and centrifuged at 1,000×g for 5 min at 4°C. The media was aspirated, and the cells were resuspended in 250 µl of lysis buffer (50 mM PIPES, pH 6.9, 50 mM NaCl, 5 mM MgCl_2_, 5 mM EGTA, 5% glycerol, 1% Triton X-100, 0.1% Tween 20, 0.1% 2-mercaptoethanol, 1 mM ATP, 1 mM phenylmethanesulfonyl fluoride, 5 mg/mL *N*-α-*p*-tosyl-L-arginine methyl ester, 10 µg/mL *N*-*p*-tosyl-L-phenylalanine chloromethyl ketone, 80 µg/mL aprotinin, 20 µg/mL pepstatin, 20 µg/mL chymostatin) and incubated on ice for 30 min. Samples were then centrifuged at 10,000×g for 10 min at 4°C. The supernatant (G-actin) was removed, and the pellet (F-actin) was resuspended in 250 µl lysis buffer. An equal volume of 2x sodium dodecyl sulfate (SDS) sample buffer was added, and samples heated to 100°C for 5 min. Equal volumes of each sample were then electrophoresed on a 10% SDS-polyacrylamide gel and electroblotted onto a polyvinylidene fluoride membrane. The membrane was blocked in non-fat dry milk for 1 h and incubated in a 1∶500 dilution of mouse anti-β-actin antibody for 1 h, followed by incubation with a 1∶5,000 dilution of goat anti-mouse antibody conjugated to alkaline phosphatase for 1 h. Colorimetric detection was achieved with 5-bromo-4-chloro-3-indolyl phosphate/nitroblue tetrazolium as described previously [42], [43].

### Actin Polymerization

Actin was purified from rabbit muscle acetone powder as previously described [44], [45], with two additional rounds of polymerization-depolymerization. Purity was confirmed by SDS-polyacrylamide gel electrophoresis. The actin was labeled to ∼70% with *N*-(1-pyrene)iodoacetamide (Invitrogen) and used in a pyrene-actin polymerization assay essentially as previously reported [46], [47]. The pyrene-G-actin was stored in 2 mM Tris, pH 8.0, 0.1 mM CaCl_2_, 0.2 mM ATP, 0.5 mM dithiothreitol (DTT). Immediately prior to the experiment, ME buffer, consisting of 50 mM MgCl_2_, 0.2 mM ethylene glycol-bis(2-aminoethyl ether)-*N*,*N*,*N′*,*N′*-tetraacetic acid (EGTA), was added to concentration of 1× from a 10× stock of ME buffer to exchange Ca^2+^ for Mg^2+^. The sample was incubated in the presence or absence of 50 µM cucurbitacin I for 1 h at 4°C, with pyrene-G-actin at a final concentration of 2 µM. Polymerization was then initiated by addition of a 1/10 volume of 10× KMEI buffer (10× = 500 mM KCl, 10 mM MgCl_2_, 10 mM EGTA, 100 mM imidazole, pH 7.0). Fluorescence intensity (340 nm excitation/420 nm emission) was measured over time on a fluorescence microplate reader (BioTek FLx800TBI).

### Actin Depolymerization

Lyophilized pyrene-labeled rabbit muscle actin (∼50% pyrene labeled from Cytoskeleton) was resuspended in water and then diluted to 23 µM in 5 mM Tris, pH 8.0, 0.2 mM CaCl_2_, 0.2 mM ATP. Pyrene-G-actin was polymerized to pyrene-F-actin according to the manufacturer's instructions by addition of a 1/25 volume of 10× actin polymerization buffer (10× = 500 mM KCl, 20 mM MgCl_2_, 10 mM ATP). The sample was stored in the dark at room temperature for 1 h. Depolymerization was initiated by diluting the pyrene-F-actin 25-fold with 5 mM Tris, pH 8.0, 0.2 mM CaCl_2_, 0.2 mM ATP, in the presence or absence of cucurbitacin I or phallacidin at the concentrations indicated in Fig. 10B. Fluorescence intensity (340 nm excitation/420 nm emission) measurements were taken over time on a fluorescence microplate reader (BioTek FLx800TBI).

As an alternative method to measure actin depolymerization, actin seeds were formed by incubating 4 µM human non-muscle G-actin (Cytoskeleton) in ISAP buffer (40 mM PIPES, pH 7.0, 2 mM DTT, 2 mM ATP, 10 mM EGTA, 100 mM KCl, 4 mM MgCl_2_) in a total volume of 200 µL overnight on ice. G-actin was polymerized to F-actin the next morning at room temperature for 1 h by combining 75 µL actin seeds with 75 µL of 4 µM G-actin in 1× ISAP buffer. 15 µL aliquots of F-actin were added to tubes, and then the actin was diluted by addition of 135 µl of 5 mM Tris, pH 7.8, 0.2 mM CaCl_2_, 0.2 mM ATP to initiate depolymerization in the presence or absence of 200 nM jasplakinolide or cucurbitacin I. Tubes were incubated overnight at 4°C. Samples were centrifuged in an airfuge for 1 h, and pellets were resuspended in 20 µL of SDS sample buffer. Samples were denatured at 100°C for 5 min and then loaded onto a 10% SDS-polyacrylamide gel. The gel was stained with GelCode Blue G-250 (Thermo Scientific Pierce) and destained in deionized water. Bands were quantified with ImageJ software.

### Mass Spectrometry

The possibility of covalent association of cucurbitacin I with purified human cofilin or human plasma gelsolin was examined by LC-ESI-MS. 15 µM of each protein was incubated with 100 µM cucurbitacin I for 6 h at 37°C. Each sample, as well as additional non-treated protein controls, was loaded onto a C18 column (Hypersil GOLD, 1.9 µm, 100 mm×1.0 mm, Thermo Scientific) on a 10ADvp high-performance LC system (Shimadzu). The proteins were eluted with a linear gradient from 2% acetonitrile/water/0.1% acetic acid to 90% acetonitrile/water/0.1% acetic acid over 20 min at a flow rate of 50 µL/min. The effluent was fed into the ESI source of a QStar Elite mass spectrometer (Applied Biosystems/MDS Sciex), with typical source conditions of IS 5500V, GS1 20, DP 80 V, FP 280 V, DP2 15 V. Acquisition of data was controlled with Analyst software (AB SCIEX).

## Supporting Information

Figure S1 Neither cofilin nor gelsolin are alkylated by cucurbitacin I. Mass spectra of human cofilin and human plasma gelsolin following incubation of 15 µM of each protein with 100 µM cucurbitacin I for 6 h at 37°C and LC-ESI-MS. The masses of the cucurbitacin I-treated proteins were identical to those of non-treated protein controls.
(1.43 MB TIF)

Movie S1 Inhibition of wound closure in MDCK cells exposed to cucurbitacin I. mCherry-actin-expressing MDCK cells were grown to confluence on a Bioptechs dish and the monolayer was wounded. The wound edge was imaged at 37°C every 5 min for 5 h in the absence of compound, and then the medium was changed to medium containing 200 nM cucurbitacin I, and the wound edge was imaged for an additional 19 h.
(6.52 MB MOV)

Movie S2 Inhibition of B16-F1 motility by cucurbitacin I. B16-F1 cells were grown overnight on laminin-coated dishes and then imaged at 37°C by phase-contrast microscopy. Images were captured every 3 min for 45 min and then continued following addition of cucurbitacin I to 100 nM for an another 45 min. The cells can be seen to rapidly stop movement and retract protrusions. The compound was then washed out and the cells were imaged over a 5-h period during which movement gradually resumed.
(1.89 MB MOV)

Movie S3 B16-F1 cells exposed to cucurbitacin I immediately stop protrusion and begin retraction of lamellae. B16-F1 cells were imaged at 37°C every 30 s for 3 h by phase-contrast microscopy and then cucurbitacin I was added to 200 nM while imaging. The movie shows the 8 min prior to compound addition and the next 40 min in the presence of compound. The freezing of protrusion is evident within a minute after addition of compound, while retraction took place over a longer time scale.
(6.03 MB MOV)

Movie S4 Cucurbitacin I does not affect the motility of *Dictyostelium* amoebae. *Dictyostelium discoidium* (strain NC4A2) were plated for 1 h in HL5 medium on a Petri dish and then imaged every 15 s at room temperature. After 1 h, cucurbitacin I was added to 200 nM, and the cells were imaged for 1.5 h. The concentration of cucurbitacin I was then raised to 2 µM and imaging was continued for another 1.5 h.
(5.15 MB MOV)

Movie S5 F-actin formation in the presence of cucurbitacin I in low-density MDCK cell cultures. MDCK cells expressing mCherry-actin were seeded on a laminin-coated dish at low density and allowed to attach. The cells were imaged at 37°C by DIC and epifluorescence every 1 min before and after the addition of 200 nM cucurbitacin I. The movie begins just after the addition of the compound to the cells. The formation of aggregates of actin over the following 5 h can be seen.
(13.96 MB MOV)

Movie S6 Jasplakinolide causes cessation of migration and lamellipodial collapse in B16-F1 cells. B16-F1 cells were grown on a laminin-coated dish overnight. Cells were then imaged at 37°C every 5 min before and after addition of 200 nM jasplakinolide. After addition, the cells immediately stopped moving and began to retract their lamellipodia. After 100 min, the compound was washed out and imaging continued. The cells recovered and began to move about 4 h later.
(0.78 MB MOV)
