# Supplementary figures and images for: Cucurbitacin I Inhibits Cell Motility by Indirectly Interfering with Actin Dynamics
Source: PLoS One. 2010 Nov 24;5(11):e14039. doi: 10.1371/journal.pone.0014039 (PMC2991314; doi:10.1371/journal.pone.0014039)

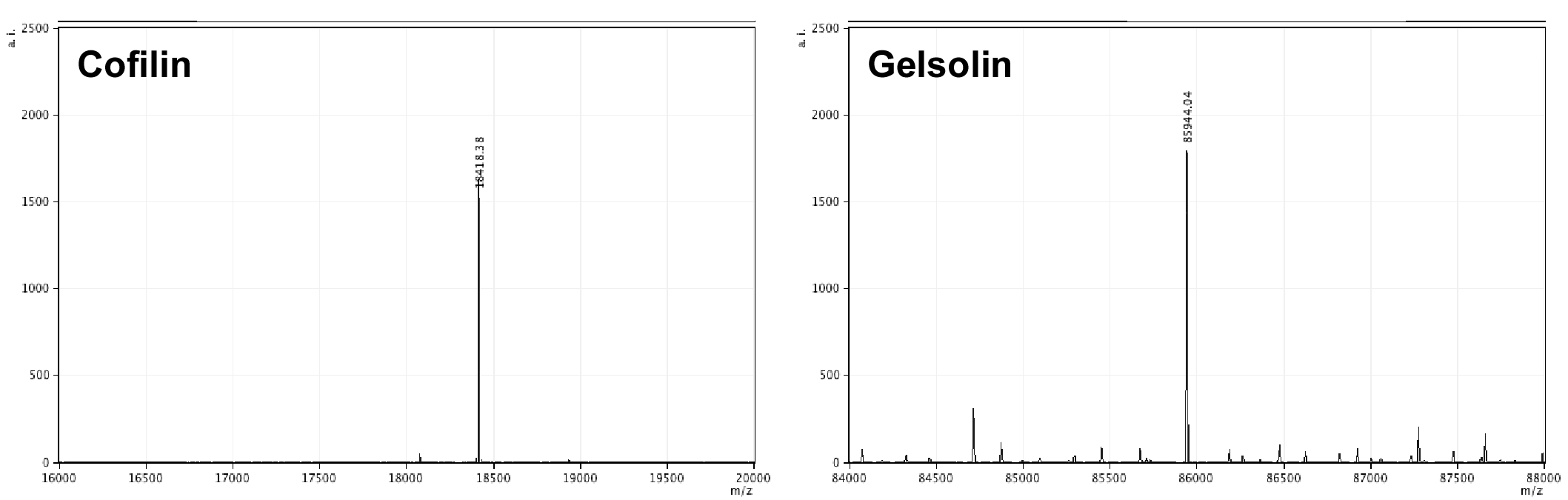

Supplement: Figure S1 — Neither cofilin nor gelsolin are alkylated by cucurbitacin I. Mass spectra of human cofilin and human plasma gelsolin following incubation of 15 µM of each protein with 100 µM cucurbitacin I for 6 h at 37°C and LC-ESI-MS. The masses of the cucurbitacin I-treated proteins were identical to those of non-treated protein controls. (1.43 MB TIF) [file pone.0014039.s001.tif]
